# Emotional reactivity to daily events in adolescents with clinical depression and subthreshold depression: an experience sampling study

**DOI:** 10.1192/j.eurpsy.2024.1127

**Published:** 2024-08-27

**Authors:** T. C.-Y. Liu, N. T.-K. Kwok, P. W.-L. Leung, S. F. Hung, S. M. S. Chan, S. L. Ma, S. W. H. Chau, W. H. Wong, S. H.-W. So

**Affiliations:** ^1^The Chinese University of Hong Kong, Hong Kong, Hong Kong; ^2^Department of Psychology; ^3^Department of Psychiatry, The Chinese University of Hong Kong, Hong Kong, Hong Kong

## Abstract

**Introduction:**

Adolescents with depression have distinct affective reactions to daily events, but current research is controversial. The emotional context insensitivity theory suggests blunted reactivity in depression, whereas the hypotheses of negative potentiation and mood brightening effect suggest otherwise. While nonlinear associations between depression severity and affective reactivity have been observed, studies with a separate subclinical group remain rare. Subthreshold depression (SD), defined by two to four symptoms lasting for two weeks or more, provides a dimensional view to the underpinnings of affective reactivity. In this study, we compared positive affect (PA) and negative affect (NA) reactivity to positive and negative daily events (uplifts and stress) among adolescents with Major Depressive Disorder (MDD), SD and healthy controls (HC) using experience sampling methods (ESM).

**Objectives:**

We hypothesized a stepped difference in affective reactivity along the depression spectrum: the MDD group will have the strongest reactivity of PA and NA to uplifts and stress, followed by SD and HC.

**Methods:**

Three groups (MDD, SD, and HC) of adolescents were recruited from an epidemiologic sample entitled ‘Hong Kong Child and Adolescent Psychiatric Epidemiologic Survey: Age 6 to 17’. Group status was determined by the Diagnostic Interview Schedule for Children Version 5. They completed an experience sampling diary on smartphone for 14 consecutive days, with 5-10 entries per day. Momentary levels of PA (happy, relaxed, contented), NA (irritated, low, nervous), uplifts and stress experienced before the entry were measured on a 1-7 Likert scale.

**Results:**

The sample consisted of 19 adolescents with MDD, 30 with SD, and 59 HC. The M:F ratio was 17:19. The age range was 12-18 with a mean of 14.8. The overall ESM completion rate was 46%. The MDD group had the highest levels of stress and NA, and the lowest levels of uplifts and PA, followed by the SD and HC groups respectively (p<0.01). Across groups, levels of PA were positively associated with uplifts and negatively associated with stress, whereas levels of NA were positively associated with stress and negatively associated with uplifts. The Group x Uplift interaction effect on PA was significant, with greater PA reactivity in SD (p<0.01) and MDD (p=0.07) when compared with HC. The Group x Uplift interaction effect on NA was significant, with greater NA reactivity in SD than HC (p<0.01). The Group x Stress interaction effect on PA was significant, with greater PA reactivity in SD than HC (p<0.01) and MDD (p<0.01). The Group x Stress interaction effect with NA is non-significant.

**Conclusions:**

Contrary to our hypothesis, adolescents with SD experienced strongest PA and NA reactivity in uplifts and PA reactivity in stress. It provides evidence towards a nonlinear relationship between severity of depression and affective reactivity.

**Disclosure of Interest:**

None Declared

